# Canonical Divergence for Flat *α*-Connections: Classical and Quantum

**DOI:** 10.3390/e21090831

**Published:** 2019-08-25

**Authors:** Domenico Felice, Nihat Ay

**Affiliations:** 1 Max Planck Institute for Mathematics in the Sciences, Inselstrasse 22, 04103 Leipzig, Germany; 2 Santa Fe Institute, 1399 Hyde Park Rd, Santa Fe, NM 87501, USA; 3 Faculty of Mathematics and Computer Science, University of Leipzig, PF 100920, 04009 Leipzig, Germany

**Keywords:** information geometry, quantum relative entropy, alpha connections, canonical divergence, Kullback-Leibler divergence

## Abstract

A recent canonical divergence, which is introduced on a smooth manifold M endowed with a general dualistic structure $\left(g,\nabla ,\nabla^{*}\right)$, is considered for flat α-connections. In the classical setting, we compute such a canonical divergence on the manifold of positive measures and prove that it coincides with the classical α-divergence. In the quantum framework, the recent canonical divergence is evaluated for the quantum α-connections on the manifold of all positive definite Hermitian operators. In this case as well, we obtain that the recent canonical divergence is the quantum α-divergence.

## 1. Introduction

Methods of Information Geometry (IG) [1] are ubiquitous in physical sciences and encompass both classical and quantum systems [2]. The natural object of study in IG is a quadruple $\left(M,g,\nabla ,\nabla^{*}\right)$ given by a smooth manifold M, a Riemannian metric g, and a pair of affine connections on M, which are dual with respect to g,

(1) $$X\;g\left(Y,Z\right)\;=\;g\left(\nabla_{X}Y,Z\right)+g\left(Y,\nabla_{X}^{*}Z\right)\;,$$

for all sections X,Y,Z∈T(M). The quadruple $\left(M,g,\nabla ,\nabla^{*}\right)$ is called a statistical manifold whenever the dual connections are both torsion-free [3]. Actually, the notion of the statistical manifold, introduced by Lauritzen [4], is usually referred to the triple (M,g,T), where $T\left(X,Y,Z\right)=g\left(\nabla_{X}^{*}Y-\nabla_{X}Y,Z\right)$ is a three-symmetric tensor. However, when ∇ and $\nabla^{*}$ are both torsion-free connections, the structures $\left(M,g,\nabla ,\nabla^{*}\right)$ and (M,g,T) are equivalent [3]. When M is a manifold of probability distributions, $g\equiv g^{F}$ is the Fisher metric, and $\nabla \equiv \nabla^{\left(e\right)}$ and $\nabla^{*}\equiv \nabla^{\left(m\right)}$ are the exponential and mixture connections [5], IG has been successfully applied to many fields, such as statistical inference, control systems theory, and neural networks (see [6] and the references therein for a comprehensive literature on applications of IG).

The geometric structure of a statistical manifold is encoded by a smooth function D:M×M→R such that:

(2) D(p,q)≥0,andD(p,q)=0iffp=q,

for all p,q∈M [7]. The dualistic structure $\left(g,\nabla ,\nabla^{*}\right)$ of M is then recovered in the following way:

(3) $$\begin{array}{cc} & g_{ij}\left(p\right)=-{\left.\partial_{i}\partial_{j}^{\prime }D\left(\xi_{p},\xi_{q}\right)\right|}_{p=q}={\left.\partial_{i}^{\prime }\partial_{j}^{\prime }D\left(\xi_{p},\xi_{q}\right)\right|}_{p=q} \end{array}$$

(4) $$\begin{array}{cc} & \Gamma_{ijk}\left(p\right)=-{\left.\partial_{i}\partial_{j}\partial_{k}^{\prime }D\left(\xi_{p},\xi_{q}\right)\right|}_{p=q},\;\Gamma_{ijk}^{*}\left(p\right)=-{\left.\partial_{i}^{\prime }\partial_{j}^{\prime }\partial_{k}D\left(\xi_{p},\xi_{q}\right)\right|}_{p=q}\;. \end{array}$$

Here,

$$\partial_{i}=\frac{\partial }{\partial \xi_{p}^{i}}\;\mathrm{and}\;\partial_{i}^{\prime }=\frac{\partial }{\partial \xi_{q}^{i}}$$

and $\left\{\xi_{p}:=\left(\xi_{p}^{1},\ldots ,\xi_{p}^{n}\right)\right\}$ and $\left\{\xi_{q}:=\left(\xi_{q}^{1},\ldots ,\xi_{q}^{n}\right)\right\}$ are local coordinate systems of p and q, respectively. Moreover, $\Gamma_{ijk}=g\left(\nabla_{\partial_{i}}\partial_{j},\partial_{k}\right)$, $\Gamma_{ijk}^{*}=g\left(\nabla_{\partial_{i}}^{*}\partial_{j},\partial_{k}\right)$ are the symbols of the dual connections ∇ and $\nabla^{*}$, respectively. The function D is called a divergence or contrast function of the statistical manifold $\left(M,g,\nabla ,\nabla^{*}\right)$ [8].

The function D is called a flat divergence if the dualistic structure $\left(g,\nabla ,\nabla^{*}\right)$ introduced on a smooth manifold M by Equations (3) and (4) is flat, namely the curvature tensors R(∇) and $R^{*}\left(\nabla^{*}\right)$ are zero. In this particular case, an attempt to connect IG with physics was established in [9]. There, the connection between IG and integrable dynamical systems was bridged by a divergence, which is canonical in some sense. More precisely, for q∈M, the gradient flows $\dot{\xi }=-{\mathrm{grad}}_{\xi }\;D\left(\xi_{q},\cdot \right)$ and $\dot{\xi }=-{\mathrm{grad}}_{\xi }\;D\left(\cdot ,\xi_{q}\right)$ converge to the point q along the $\nabla^{*}$-geodesic and the ∇-geodesic, respectively. In this context, when M is the manifold of Gaussian distributions with mean μ and variance $\sigma^{2}$, the Kullback-Leibler (KL) divergence induces a dualistic structure given by the Fisher metric and the (e),(m) connections. In this case, the dynamics of the above-mentioned gradient flows, given now in terms of the KL divergence, turns out to be the Uhlenbeck-Ornstein process $\mu =\mu_{0}+v\left(t+\tau \right)\;,\;\;\sigma^{2}=2\;D\left(t+\tau \right)$ characterized by the drift coefficient v and diffusion coefficient D [9]. Further connections between IG and dynamical systems can be found in [10], where certain gradient flows on Gaussian and multinomial distributions are characterized as completely integrable Hamiltonian systems.

The Kullback-Leibler divergence has been effectively employed to quantify the complexity of a system described by a probability distribution p in terms of its deviation from an exponential family of probability distributions [11]. The quantum version of the KL divergence, namely the quantum relative entropy, induces on the manifold of quantum states a dually flat structure given by a quantum version of the Fisher metric tensor and two flat connections, also called the mixture and the exponential connections, which are dual in the sense of Equation (1) [12]. Moreover, the quantum relative entropy has been used to quantify the many-party correlations of a composite quantum state ρ as the deviation of it from a Gibbs family of quantum states [13]. The utility of the quantum relative entropy as a measure of the complexity for quantum states was shown in [14], where algorithms for its evaluation were studied. Furthermore, in that context, the many-party correlations were related to the entanglement of quantum systems as defined in [15].

A generalization of the flat structures induced by the Kullback-Leibler divergence on the finite classical systems and by the quantum relative entropy on the finite quantum states is provided by the classical α-divergence and the quantum α-divergence, respectively. Both generate a one-parameter family of connections, the α-connections, which are dual with respect to the Fisher metric in the classical case [1], whereas in the quantum case, they are dual with respect to the Wigner-Yanase-Dyson metric [16].

From a physical viewpoint, it is worth remarking that the Boltzmann-Gibbs distribution in statistical physics is an exponential family such that an invariant flat structure is given to the underlying manifold in terms of the (e)-connection [6]. Tsallis generalized the concept of the Boltzmann-Gibbs distribution by introducing a generalized entropy, called the q-entropy, for studying various phenomena not included in the conventional Boltzmann-Gibbs framework [17,18]. Actually, the α-geometry, which is induced by the α-divergence, covers the geometry of q-entropy physics [19]. Therefore, the α-divergence can be understood as a generalization of the classical KL divergence also from a physical standpoint. In the quantum case, the geometry of q-entropy physics has been successfully employed for carrying out a criterion that detects the critical frontier, which has separable states on one side and quantum entangled ones on the other [20].

The purpose of the present article is to consider a recent canonical divergence, introduced by Ay and Amari in [21], as a tool for unifying classical and quantum information geometry. In particular, we aim to prove that this canonical divergence is the classical α-divergence when computed on the space of positive measures, as well as the quantum α-divergence if evaluated on the manifold of positive definite Hermitian operators.

## 2. Canonical Divergence and the Inverse Problem in Information Geometry

The inverse problem within information geometry concerns the search for a divergence function D, which recovers a given dual structure $\left(g,\nabla ,\nabla^{*}\right)$ of a smooth manifold M according to Equations (3) and (4). The solution to this problem was provided by Matumoto, who showed that such a divergence always exists for any statistical manifold [22]. Nonetheless, this solution is not unique, and infinitely many divergences can be defined on M, which gives the same dualistic structure. For this reason, seeking a divergence that can be considered as the most natural is of utmost importance. To this end, Amari and Nagaoka defined a Bregman-type divergence on dually flat manifolds [1]. This one has relevant properties such as the generalized Pythagorean theorem and the geodesic projection theorem, and it is commonly assessed as the natural solution of the inverse problem in information geometry for dually flat manifolds. This is exactly why the Amari and Nagaoka divergence is referred to as the canonical divergence of dually flat statistical manifolds. However, the need for a general canonical divergence, which applies to any dualistic structure, is a very crucial issue, as pointed out in [23]. According to the theory developed in [3], a divergence function D of a statistical manifold $\left(M,g,\nabla ,\nabla^{*}\right)$ is called canonical if:

- D generates the dualistic structure $\left(g,\nabla ,\nabla^{*}\right)$ based on Equations (3) and (4);
- D is one half of the squared Riemannian distance, i.e., $D\left(p,q\right)=\frac{1}{2}\;d{\left(p,q\right)}^{2}$, when the statistical manifold is self-dual, namely when $\nabla =\nabla^{*}$ coincides with the Levi–Civita connection of g;
- D is the canonical divergence of the Bregman type when $\left(M,g,\nabla ,\nabla^{*}\right)$ is dually flat.

Ay and Amari recently defined a divergence for a general dualistic structure in terms of geodesic integration of the inverse exponential map [21]. It turns out that such a divergence satisfies all the above-mentioned requirements. Therefore, it can be viewed as a canonical divergence for a general dualistic structure. For p,q∈M, consider the ∇-geodesic $\tilde{\gamma }\left(t\right)\;\left(0\leq t\leq 1\right)$ connecting q with p, the recent canonical divergence introduced in [21] is then defined by:

(5) $$D\left(p,q\right):=\int_{0}^{1}{\langle X_{t}\left(p\right),\dot{\tilde{\gamma }}\left(t\right)\rangle }_{\tilde{\gamma }\left(t\right)}\;dt\;,\;X_{t}\left(p\right):=\exp_{\tilde{\gamma }\left(t\right)}^{-1}\left(p\right)\;.$$

The ∇-exponential map, exp:TM→M, is defined by $\exp\left(X\right)=\gamma_{X}\left(1\right)$ whenever the ∇-geodesic $\gamma_{X}\left(t\right)$, satisfying ${\dot{\gamma }}_{X}\left(0\right)=X$, exists on an interval of t containing [0,1]. Therefore, if γ:[0,1]→M is the ∇-geodesic such that γ(0)=p and γ(1)=q, the inverse at p of the exponential map is given by $\exp_{p}^{-1}\left(q\right):=\dot{\gamma }\left(0\right)$. According to this definition, for every t∈[0,1], we can consider the ∇-geodesic $\gamma_{t}\left(s\right)$ such that $\gamma_{t}\left(0\right)=p$ and $\gamma_{t}\left(1\right)=\gamma \left(t\right)$ and then define the ∇-velocity vector at p by $X_{p}\left(\gamma \left(t\right)\right):=\exp_{p}^{-1}\left(\gamma \left(t\right)\right)={\dot{\gamma }}_{t}\left(0\right)$. In this way, the vector field $X_{t}\left(p\right)$ of Equation (5) turns out to be given by $X_{t}\left(p\right)=P_{\gamma (t)}\;X_{p}\left(\gamma \left(t\right)\right)=t\;\dot{\gamma }\left(t\right)$. Here, $P:T_{p}M\to T_{\gamma (t)}M$ is the ∇-parallel transport from p to γ(t). In light of all this, the divergence D(p,q) assumes the following useful expression:

(6) $$D\left(p,q\right)=\int_{0}^{1}\;t\;{∥\dot{\gamma }\left(t\right)∥}^{2}\;dt\;.$$

Analogously, the dual function of D(p,q) is defined as the $\nabla^{*}$-geodesic integration of the inverse of the $\nabla^{*}$-exponential map [21]. Therefore, we have for the dual divergence $D^{*}$ a similar expression as Equation (6) for the canonical divergence D:

(7) $$D^{*}\left(p,q\right)=\int_{0}^{1}\;t\;{∥{\dot{\gamma }}^{*}\left(t\right)∥}^{2}\;dt\;,$$

where $\gamma^{*}\left(t\right)\;\left(0\leq t\leq 1\right)$ is the $\nabla^{*}$-geodesic connecting p with q.

The canonical divergence given by Equation (6) has been recently proposed as a tool for unifying classical and quantum information geometry [24]. In particular, it has been considered on the simplex of probability measures:

(8) $$S:=\left\{p=\sum_{i}\;p_{i}\;\delta_{i}\in R^{n}\;|\;p_{i}>0\;\text{for all}\;i,\;\mathrm{and}\;\sum_{i}\;p_{i}=1\right\}\;,$$

as well as on the manifold of quantum finite states,

(9) $$S:=\left\{\rho :H\to C\;|\;\rho =\rho^{†}>0,\;\mathrm{Tr}\rho =1\right\}\;,$$

where H denotes a finite-dimensional Hilbert space and ρ is any Hermitian operator on H. The natural dualistic structure on the simplex S is given, in classical information geometry, in terms of the Fisher metric $g^{F}$ and two flat connections, the mixture $\nabla^{\left(m\right)}$ and the exponential $\nabla^{\left(e\right)}$ ones, which are dual with respect to $g^{F}$ in the sense of Equation (1) [5]. Very remarkably, the Fisher metric is the only monotone Riemannian metric (up to a positive factor) on the class of finite probability simplices [25]. The quantum version of a monotone Riemannian metric on the manifold of quantum finite states S is given in terms of the notion of stochastic mapping [26]. More precisely, let A be the set of all Hermitian operators on the Hilbert space H; a linear mapping T:A→A is said to be stochastic if T(S)⊂S and T is completely positive [27]. Furthermore, a family $\left\{{\langle \langle \;,\;\rangle \rangle }_{\rho }\;|\;\rho \in S\right\}$ of inner products on A is said to be monotone if ${\langle \langle A,A\rangle \rangle }_{\rho }\geq {\langle \langle T\left(A\right),T\left(A\right)\rangle \rangle }_{T(\rho )}$ for any arbitrary stochastic map T and for every ρ∈S [26]. Due to Petz, there are infinitely many monotone inner products on H. Therefore, the quantum analogue of the Fisher metric is not unique [28]. However, when the flat connections are required to be torsion-free, a natural dualistic structure on the manifold of quantum states S is the one induced by the Bogoliubov-Kubo-Mori (BKM) inner product [12]. Furthermore, it turns out that the only monotone metrics that make the mixture connection $\nabla^{\left(m\right)}$ and the exponential connection $\nabla^{\left(e\right)}$ dual are the scalar multiples of the BKM metric [29]. When the canonical divergence (6) is computed on the simplex S, it is shown to be the Kullback-Leibler divergence,

(10) $$D\left(p,q\right)\;=\;\sum_{i=1}^{n}\;p_{i}\;\log\left(\frac{p_{i}}{q_{i}}\right)\;,\;p,\;q\;\in S\;,$$

which proves that D(p,q) recovers the natural dualistic structure on S given by the Fisher metric $g^{F}$ and the dually flat connections $\nabla^{\left(m\right)}$ and $\nabla^{\left(e\right)}$ [24]. Analogously, when D is considered on the manifold of quantum states S, it has been proven that:

(11) $$D\left(\rho ,\sigma \right)=\mathrm{Tr}\;\rho \left(\log\rho -\log\sigma \right)\;,\;\rho ,\;\sigma \;\in S\;,$$

where Tr denotes the trace operator on the finite-dimensional Hilbert space of density matrices [24]. The function on the right-hand side of Equation (11) is called the quantum relative entropy, and it recovers the dual structure of S given by the metric induced by the BKM inner product and the flat connections $\nabla^{\left(m\right)}$ and $\nabla^{\left(e\right)}$ [1].

In this article, we aim to investigate the canonical divergence (6) on the manifold of positive measures, as well as on the space of positive definite Hermitian operators for the more general α-connections. In the classical information geometry, the one-parameter family of the α-connections is defined on the manifold of positive measures by the linear combination of the mixture and exponential connections [6],

(12) $$\nabla^{\alpha }\;=\;\frac{1-\alpha }{2}\;\nabla^{\left(m\right)}+\frac{1+\alpha }{2}\;\nabla^{\left(e\right)}\;.$$

It turns out that $\nabla^{\alpha }$ and $\nabla^{-\alpha }$ are dual with respect to the Fisher metric $g^{F}$ in the sense of Equation (1). In quantum information geometry, α-connections appeared also in terms of the Amari α-embeddings [30]. While in the classical information geometry, the two definitions coincide, this is no longer true in quantum information geometry [31]. In the present paper, we consider the definition of the quantum α-connections by means of the Amari α-embedding, which excludes that they can be obtained by the convex mixture of $\nabla^{\left(m\right)}$ and $\nabla^{\left(e\right)}$ connections. The natural inner product that makes the quantum α-connections dual in the sense of Equation (1) is the Wigner-Yanase-Dyson (WYD) metric, which appeared for the first time in the context of quantum information geometry in the work of Hasegawa [32]. Actually, it turns out that this is the only monotone metric (up to a scalar multiple) that makes the quantum $\nabla^{\alpha }$ and $\nabla^{-\alpha }$ dual [16].

## 3. Classical Flat Alpha-Divergence

We represent measures on the set {1,…,n} as elements of $R^{n}$. In this representation, the Dirac measures $\delta_{i},\;i=1,\ldots ,n$ form the canonical basis of $R^{n}$. The n-dimensional cone of positive measures on the set {1,…,n} is then defined by:

(13) $$M_{+}:=R_{+}^{n}=\left\{p=\sum_{i=1}^{n}\;p_{i}\;\delta_{i}\in R^{n}\;|\;p_{i}>0\;\;\text{for all}\;\;i\right\}\;.$$

The very natural Riemannian metric on $M_{+}$ is the Fisher metric [6], which is defined by:

(14) $$g_{p}^{F}\left(X,Y\right)=\sum_{i=1}^{n}\;\frac{1}{p_{i}}\;X_{i}\;Y_{i}\;,$$

for all $p\in M_{+}$ and $X,Y\in T_{p}M_{+}$. Here, $T_{p}M_{+}$ denotes the tangent space to $M_{+}$ at p. Given the mixture and the exponential connections, we can define the α-connection on $M_{+}$ by using Equation (12). Recalling that $T_{p}M_{+}\equiv R^{n}$, we can write any $X\in T_{p}M_{+}$ with respect to the canonical basis of $R^{n}$. Hence, the (m)-connection on $M_{+}$ reads as follows:

(15) $${\left.\nabla_{X}^{\left(m\right)}Y\right|}_{p}=\sum_{i=1}^{n}\frac{\partial Y_{i}}{\partial X}\left(p\right)\;\delta_{i}\;,$$

for all vector fields $X=(X_{1},\ldots ,X_{n})$ and $Y=(Y_{1},\ldots ,Y_{n})$ on $M_{+}$. Here, ∂/∂X denotes the derivative in the direction $X\in T_{p}M_{+}$. On the contrary, the (e)-connection is given by:

(16) $${\left.\nabla_{X}^{\left(e\right)}Y\right|}_{p}=\sum_{i=1}^{n}\left(\frac{\partial Y_{i}}{\partial X}\left(p\right)-\frac{1}{p_{i}}\;X_{i}\;Y_{i}\right)\;\delta_{i}\;.$$

Therefore, by applying Equation (12), we can describe the α-connection for the manifold $M_{+}$ as follows,

(17) $$\begin{array}{ccc} {\left.\nabla_{X}^{\alpha }Y\right|}_{p} & = & \frac{1-\alpha }{2}{\left.\nabla_{X}^{\left(m\right)}Y\right|}_{p}+\frac{1+\alpha }{2}{\left.\nabla_{X}^{\left(e\right)}Y\right|}_{p}\\ & = & {\left.\nabla_{X}^{\left(m\right)}Y\right|}_{p}+\frac{1+\alpha }{2}\left({\left.\nabla_{X}^{\left(e\right)}Y\right|}_{p}-{\left.\nabla_{X}^{\left(m\right)}Y\right|}_{p}\right)\\ & = & \sum_{i=1}^{n}\left(\frac{\partial Y_{i}}{\partial X}\left(p\right)-\frac{1+\alpha }{2}\frac{X_{i}\;Y_{i}}{p_{i}}\right)\;\delta_{i}\;, \end{array}$$

for all vector fields X,Y on $M_{+}$. It turns out that the dualistic structure $\left(g^{F},\nabla^{\alpha },\nabla^{-\alpha }\right)$ is dually flat, i.e., the Riemannian curvature tensors of $\nabla^{\alpha }$ and $\nabla^{-\alpha }$ are zero [6]. Furthermore, very naturally, such an α-flat dualistic structure is induced on $M_{+}$ by the α-divergence $D^{\left(\alpha \right)}$, which is commonly assessed as the canonical solution of the inverse problem for recovering the dually flat α-structure $\left(g^{F},\nabla^{\alpha },\nabla^{-\alpha }\right)$ on $M_{+}$ [6]. Given $p,\;q\in M_{+}$, the α-divergence between p and q is given by:

(18) $$D^{\left(\alpha \right)}\left(p,q\right)\;=\;\sum_{i=1}^{n}\;\left(\frac{2}{1-\alpha }\;q_{i}+\frac{2}{1+\alpha }\;p_{i}-\frac{4}{1-\alpha^{2}}\;q_{i}^{\frac{1+\alpha }{2}}p_{i}^{\frac{1-\alpha }{2}}\right)\;.$$

The flat α-divergence (18) has been deeply studied, and its features have been widely discussed in the literature (see [3,6] for more details). In particular, we point out that $D^{\left(\alpha \right)}$ is a continuous function of the parameter α, and the limit α→−1 gives the well-known Kullback-Leibler divergence on the manifold of positive measures,

(19) $$\lim_{\alpha \to -1}D^{\left(\alpha \right)}\left(p,q\right)=\sum_{i=1}^{n}\left(q_{i}-p_{i}-p_{i}\log\frac{q_{i}}{p_{i}}\right)\;,\;p,q\in M_{+}\;,$$

whereas the limit α→+1 gives:

$$\lim_{\alpha \to +1}D^{\left(\alpha \right)}\left(p,q\right)=\sum_{i=1}^{n}\left(p_{i}-q_{i}-q_{i}\log\frac{p_{i}}{q_{i}}\right)\;,\;p,q\in M_{+}\;.$$

Let us observe that, if we restrict (19) to the simplex of probability distributions, namely when $\sum_{i=1}^{n}\;p_{i}=\sum_{i=1}^{n}\;q_{i}=1$, then we obtain the function (10). At this point, it is worth mentioning the close connection between the α-divergence (18) and the Tsallis relative entropy, or q-divergence, on the manifold of positive measures. The q-divergence on $M_{+}$ is defined by (see [33] for more details):

(20) $$D_{q}\left(p,q\right)=\frac{1}{1-q}\sum_{i=1}^{n}\left(q\;p_{i}+\left(1-q\right)\;q_{i}-p_{i}^{q}\;q_{i}^{1-q}\right)\;,\;p,q\in M_{+}\;.$$

By setting α=1−2q, we can easily verify that the flat α-divergence (18) and the Tsallis q-divergence (20) coincide up to a scaling factor. Further, the limit q→1, that is α→−1, recovers the Kullback-Leibler divergence,

$$\lim_{q\to 1}\;D_{q}\left(p,q\right)=\lim_{\alpha \to -1}\;\frac{1-\alpha }{2}\;D^{\left(\alpha \right)}\left(p,q\right)=\sum_{i=1}^{n}\left(q_{i}-p_{i}-p_{i}\log\frac{q_{i}}{p_{i}}\right)\;,\;p,q\in M_{+}\;.$$

In this section, we aim to compute the canonical divergence (6) on $M_{+}$ for the α-connections given by (17). In order to achieve this result, we need to consider the geodesic with respect to the $\nabla^{\alpha }$-connection. Let $p,\;q\in M_{+}$, a curve $\gamma :\left[0,1\right]\to M_{+}$ from p to q is an α-geodesic iff $\nabla_{\dot{\gamma }}^{\alpha }\dot{\gamma }=0$ and γ(0)=p, γ(1)=q. From Equation (17), we then obtain the following geodesic equations:

(21) $${\ddot{\gamma }}_{i}-\frac{1+\alpha }{2}\;\frac{{\dot{\gamma }}_{i}^{2}}{\gamma_{i}}=0,\;i=1,\ldots ,n\;,\;\gamma \left(0\right)=p,\;\;\gamma \left(1\right)=q\;,$$

where $\gamma \left(t\right)=(\gamma_{1}\left(t\right),\ldots ,\gamma_{n}\left(t\right))$. Hence, the solution of (21) is the α-geodesic from p to q, and it is given by:

(22) $$\gamma^{\left(\alpha \right)}\left(t\right)={\left(\left(1-t\right)\;p^{\frac{1-\alpha }{2}}+t\;q^{\frac{1-\alpha }{2}}\right)}^{\frac{2}{1-\alpha }},\;\;\;t\in \left[0,1\right]\;.$$

At this point, we can apply Equation (6) to compute the canonical divergence D(p,q) on the manifold $M_{+}$ of positive measures. From Equation (14), we obtain:

$$\begin{array}{ccc} ∥{\dot{\gamma }}^{\left(\alpha \right)}{\left(t\right)∥}_{\gamma^{\left(\alpha \right)}\left(t\right)}^{2} & = & g_{\gamma^{\left(\alpha \right)}\left(t\right)}^{F}\left({\dot{\gamma }}^{\left(\alpha \right)}\left(t\right),{\dot{\gamma }}^{\left(\alpha \right)}\left(t\right)\right)=\sum_{i=1}^{n}\;\frac{{\left({\dot{\gamma }}_{i}^{\left(\alpha \right)}\right)}^{2}}{\gamma_{i}^{\left(\alpha \right)}}\\ & = & \sum_{i=1}^{n}\;\frac{4}{{\left(1-\alpha \right)}^{2}}\;\frac{{\left(\left(1-t\right)\;p^{\frac{1-\alpha }{2}}+t\;q^{\frac{1-\alpha }{2}}\right)}^{2\frac{1+\alpha }{1-\alpha }}}{{\left(\left(1-t\right)\;p^{\frac{1-\alpha }{2}}+t\;q^{\frac{1-\alpha }{2}}\right)}^{\frac{2}{1-\alpha }}}\;{\left(q^{\frac{1-\alpha }{2}}-p^{\frac{1-\alpha }{2}}\right)}^{2}\\ & = & \sum_{i=1}^{n}\;\frac{4}{{\left(1-\alpha \right)}^{2}}\;{\left(\left(1-t\right)\;p^{\frac{1-\alpha }{2}}+t\;q^{\frac{1-\alpha }{2}}\right)}^{\frac{2\;\alpha }{1-\alpha }}\;{\left(q^{\frac{1-\alpha }{2}}-p^{\frac{1-\alpha }{2}}\right)}^{2}\;. \end{array}$$

Now, we can compute the integral in (6) by performing an integration by parts:

$$\begin{array}{ccc} D(p,q) & = & \sum_{i=1}^{n}\;\int_{0}^{1}\;t\;\frac{4}{{\left(1-\alpha \right)}^{2}}\left[{\left(\left(1-t\right)\;p_{i}^{\frac{1-\alpha }{2}}+t\;q_{i}^{\frac{1-\alpha }{2}}\right)}^{\frac{2\;\alpha }{1-\alpha }}\;\left(q_{i}^{\frac{1-\alpha }{2}}-p_{i}^{\frac{1-\alpha }{2}}\right)\right]\;\left(q_{i}^{\frac{1-\alpha }{2}}-p_{i}^{\frac{1-\alpha }{2}}\right)\;dt\\ & = & \sum_{i=1}^{n}\{{\left[t\frac{4}{{\left(1-\alpha \right)}^{2}}\frac{1-\alpha }{1+\alpha }{\left(\left(1-t\right)\;p_{i}^{\frac{1-\alpha }{2}}+t\;q_{i}^{\frac{1-\alpha }{2}}\right)}^{\frac{1+\alpha }{1-\alpha }}\right]}_{0}^{1}\left(q_{i}^{\frac{1-\alpha }{2}}-p_{i}^{\frac{1-\alpha }{2}}\right)\\ & & -\int_{0}^{1}\;\frac{4}{{\left(1-\alpha \right)}^{2}}\frac{1-\alpha }{1+\alpha }{\left(\left(1-t\right)\;p_{i}^{\frac{1-\alpha }{2}}+t\;q_{i}^{\frac{1-\alpha }{2}}\right)}^{\frac{1+\alpha }{1-\alpha }}\left(q_{i}^{\frac{1-\alpha }{2}}-p_{i}^{\frac{1-\alpha }{2}}\right)dt\}\\ & = & \sum_{i=1}^{n}\frac{4}{1-\alpha^{2}}\left\{q_{i}^{\frac{1+\alpha }{2}}\left(q_{i}^{\frac{1-\alpha }{2}}-p_{i}^{\frac{1-\alpha }{2}}\right)-\int_{0}^{1}{\left(\left(1-t\right)\;p_{i}^{\frac{1-\alpha }{2}}+t\;q_{i}^{\frac{1-\alpha }{2}}\right)}^{\frac{1+\alpha }{1-\alpha }}\left(q_{i}^{\frac{1-\alpha }{2}}-p^{\frac{1-\alpha }{2}}\right)dt\right\}\\ & = & \sum_{i=1}^{n}\frac{4}{1-\alpha^{2}}\left(q_{i}-p_{i}^{\frac{1-\alpha }{2}}\;q_{i}^{\frac{1+\alpha }{2}}\right)-\frac{4}{1-\alpha^{2}}\frac{1-\alpha }{2}{\left[{\left(\left(1-t\right)\;p_{i}^{\frac{1-\alpha }{2}}+t\;q_{i}^{\frac{1-\alpha }{2}}\right)}^{\frac{2}{1-\alpha }}\right]}_{0}^{1}\\ & = & \sum_{i=1}^{n}\left\{\frac{4}{1-\alpha^{2}}\;q_{i}-\frac{4}{1-\alpha^{2}}\;p_{i}^{\frac{1-\alpha }{2}}\;q_{i}^{\frac{1+\alpha }{2}}-\frac{2}{1+\alpha }\;q_{i}+\frac{2}{1+\alpha }\;p_{i}\right\}\;. \end{array}$$

This proves that:

(23) $$D\left(p,q\right)\;=\;\sum_{i=1}^{n}\;\left(\frac{2}{1-\alpha }\;q_{i}+\frac{2}{1+\alpha }\;p_{i}-\frac{4}{1-\alpha^{2}}\;q_{i}^{\frac{1+\alpha }{2}}p_{i}^{\frac{1-\alpha }{2}}\right)\;,$$

that is the canonical divergence (6) coincides with the α-divergence (18) on the manifold $M_{+}$ of positive measures.

## 4. Quantum Flat Alpha-Divergence

The goal of this section is to compute the canonical divergence of Equation (6) on the manifold of positive definite Hermitian operators endowed with the quantum α-connections. In the classical case, the definition of the α-connection $\nabla^{\alpha }$ can be equivalently given by Equation (12) or by means of the well-known Amari α-embedding [6]. In the quantum setting, by exploiting the linear structure of the manifold, we can introduce the quantum mixture connection $\nabla^{\left(m\right)}$ on the space of positive definite Hermitian operators. On the other hand, the quantum exponential connection $\nabla^{\left(e\right)}$ is introduced by relying on the linear structure of logarithms of the manifold. Both connections, $\nabla^{\left(m\right)}$ and $\nabla^{\left(e\right)}$, are flat. Furthermore, they turn out to be dual in the sense of Equation (1) with respect to the metric induced by the BKM inner product [12]. Actually, this metric is (up to a scalar multiple) the only one that makes $\nabla^{\left(m\right)}$ and $\nabla^{\left(e\right)}$ dual on the manifold of positive definite Hermitian operators. A generalization of these two natural connections is provided by the quantum α-connections, which, on the manifold under consideration, are flat ones, as well. In our approach, these α-connections are introduced in terms of the Amari α-embeddings, and then, they cannot be obtained by the convex combination of the mixture and the exponential connections as in the classical setting [16]. It turns out that for α∈[−3,3], the connections $\nabla^{\alpha }$ and $\nabla^{-\alpha }$ are torsion-free and dual with respect to the WYD metric [34]. However, the target spaces of our approach are $L^{r}$, with $r=\frac{2}{1-\alpha }$, and then, we restrict our discussion to the range α∈(−1,1).

Recall that in order to compute the canonical divergence (6), we need to get the α-geodesic $\gamma_{\alpha }$ between any two positive Hermitian operators $\rho_{1}$ and $\rho_{2}$. To do this, we shall describe the α-connections in terms of the $\nabla^{\alpha }$-parallel transport, which is introduced very naturally on the manifold of positive Hermitian operators. Given B(H), the algebra of linear operators on an N-dimensional complex Hilbert space H, the subspace of Hermitian operators is an $N^{2}$-dimensional real vector space defined by:

(24) $$A:=\{\rho \in B\left(H\right)\;|\;\rho =\rho^{†}\}\;,$$

where $\rho^{†}={\bar{\rho }}^{t}$ and t, here, denotes the transpose matrix. Therefore, the manifold of all positive definite Hermitian operators, or more simply, quantum operators, is given by:

(25) $$M_{+}:=\left\{\rho \in A\;|\;\rho >0\right\}\;.$$

The α-embedding:

(26) $$l_{\alpha }:M_{+}\to A,\;l_{\alpha }\left(\rho \right):=\frac{2}{1-\alpha }\;\rho^{\frac{1-\alpha }{2}}\;,$$

maps the manifold of quantum operators into the vector space A for all α∈(−1,1) [1]. Furthermore, for $\rho \in M_{+}$, we have that $T_{\rho }M_{+}=T_{\rho }A=A$. Hence, the α-embedding supplies a useful representation of the tangent bundle of $M_{+}$. In fact, by considering the subspace of A given by:

(27) $$A^{\left(\alpha \right)}:=\left\{A\in A\;|\;\mathrm{Tr}\left(\rho^{\frac{1-\alpha }{2}}A\right)=0\right\}\;,\;\rho \in M_{+}\;,$$

we can then define the isomorphism:

(28) $${\left(l_{\alpha }\right)}_{*(\rho )}:T_{\rho }M_{+}\to A^{\left(\alpha \right)},\;{\left(l_{\alpha }\right)}_{*(\rho )}\left(X\right):={\left(l_{\alpha }\circ \gamma \right)}^{\prime }\left(0\right)\;,$$

where $\gamma :\left(-\varepsilon ,\varepsilon \right)\to M_{+}$ is a curve such that $\gamma^{\prime }\left(0\right)=X$. This isomorphism provides the α-representation of the tangent space $T_{\rho }M_{+}$ [16]. In particular, if $\left\{\xi^{1},\ldots ,\xi^{n}\right\}$ is a coordinate system for $M_{+}$, then the α-representation of the basis $\left\{\frac{\partial }{\partial \xi^{1}},\ldots ,\frac{\partial }{\partial \xi^{n}}\right\}$ of $T_{\rho }M_{+}$ is $\left\{\frac{\partial l_{\alpha }}{\partial \xi^{1}},\ldots ,\frac{\partial l_{\alpha }}{\partial \xi^{n}}\right\}$, where $n=N^{2}$. Finally, for any vector field $X\in T(M_{+})$, we have that its α-representation is defined by:

(29) $${\left(X\right)}^{\left(\alpha \right)}\left(\rho \right):={\left(l_{\alpha }\right)}_{*(\rho )}X_{\rho }\;.$$

From Equation (26), we may observe that the $l_{\alpha }\left(\rho \right)\in M_{+}$ for all $\rho \in M_{+}$. Now, since $T_{\rho }M_{+}=A$, we can simply define the α-parallel transport on $M_{+}$ by:

(30) $$\Pi_{\rho_{1},\rho_{2}}^{\left(\alpha \right)}:T_{\rho_{1}}M_{+}\to T_{\rho_{2}}M_{+},\;\Pi_{\rho_{1},\rho_{2}}^{\left(\alpha \right)}\left(X\right):={\left(l_{\alpha }\right)}_{*(\rho_{2})}^{-1}\left({\left(l_{\alpha }\right)}_{*(\rho_{1})}\left(X\right)\right)\;,\;\forall \;,\rho_{1},\rho_{2}\in M_{+}\;.$$

Therefore, we find that the α-representation of the covariant derivative $\nabla^{\alpha }$ associated with $\Pi^{\left(\alpha \right)}$ is:

(31) $${\left(\nabla_{\partial_{i}}^{\alpha }\partial_{j}\right)}^{\left(\alpha \right)}=\frac{\partial^{2}\;l_{\alpha }\left(\rho \right)}{\partial \xi^{i}\partial \xi^{j}}\;,$$

where $\left\{\xi^{1},\ldots ,\xi^{n}\right\}$ is an arbitrary coordinate system for $M_{+}$ and $n=N^{2}$ is the topological dimension of the tangent space $T_{\rho }M_{+}=A$. In order to show that $M_{+}$ is $\nabla^{\alpha }$-flat, consider a basis $\left\{A_{1},\ldots ,A_{n}\right\}$ of A. Then, there exist real numbers $\left\{\theta^{1},\ldots ,\theta^{n}\right\}$ such that we can write $\rho^{\frac{1-\alpha }{2}}\in A$ as follows:

$$\frac{2}{1-\alpha }\rho^{\frac{1-\alpha }{2}}=\theta^{1}A_{1}+\ldots +\theta^{n}A_{n}\;,$$

for every $\rho \in M_{+}$. We can see from Equation (31) that:

$${\left(\nabla_{\partial_{i}}^{\alpha }\frac{\partial }{\partial \theta^{j}}\right)}^{\left(\alpha \right)}=\frac{\partial^{2}l_{\alpha }\left(\rho \right)}{\partial \theta^{i}\partial \theta^{j}}=\frac{\partial A_{j}}{\partial \theta^{i}}=0\;.$$

This proves that $\left\{\theta^{1},\ldots ,\theta^{n}\right\}$ is a $\nabla^{\alpha }$-affine coordinate system for $M_{+}$, and then, $M_{+}$ is $\nabla^{\alpha }$-flat [16]. As a consequence, for any couple of points $\rho_{1},\rho_{2}\in M_{+}$, we can write the α-geodesic from $\rho_{1}$ to $\rho_{2}$ in these $\nabla^{\alpha }$-affine coordinates as follows:

(32) $$\gamma_{\alpha }\left(t\right)=\frac{2}{1-\alpha }\left(t\;\rho_{2}^{\frac{1-\alpha }{2}}+\left(1-t\right)\;\rho_{1}^{\frac{1-\alpha }{2}}\right)\;.$$

In order to perform the computation of the integral (6), we need to calculate the norm $∥{\dot{\gamma }}_{\alpha }{\left(t\right)∥}_{\gamma_{\alpha }\left(t\right)}^{2}$. To do this, we have to specify a suitable metric on the tangent space $TM_{+}$. As discussed above, we select the metric induced by the WYD inner product because this metric is the only one (up to a scalar multiple) that makes the quantum flat α-connections dual in the sense of Equation (1) [29]. For any X,Y vector fields on $M_{+}$, this metric turns out to be defined by:

(33) $$g_{\rho }^{\alpha }\left(X,Y\right)=\mathrm{Tr}\left(X^{\left(\alpha \right)}\;Y^{\left(-\alpha \right)}\right),\;X,Y\in T_{\rho }M_{+}\;,$$

where $X^{\left(\alpha \right)}$ denotes the α-representation of the tangent vector X, and it is given by Equation (29) [35]. It is worth noticing that the limit $\lim_{\alpha \to \pm 1}g^{\alpha }$ gives the metric induced by the BKM inner product on the manifold of density operators [12].

In order to write the WYD metric with respect to the $\nabla^{\alpha }$-affine coordinate system $\left\{\theta^{1},\ldots ,\theta^{n}\right\}$, we may observe that:

(34) $$\frac{\partial l_{\alpha }\left(\rho \right)}{\partial \theta^{i}}=A_{i}\;,$$

where $A_{i}$ is a vector of the basis $\{A_{1},\ldots ,A_{n}\}\subset A$. This implies that, with this coordinate system, the (α)-representation $X^{\left(\alpha \right)}$ of a vector field $X\in T(M_{+})$ is the vector field X itself. In addition, we also have that:

(35) $$l_{-\alpha }=\frac{1+\alpha }{2}\rho^{\frac{1+\alpha }{2}}=\left(\frac{2}{1+\alpha }\right){\left(\frac{1-\alpha }{2}\right)}^{\frac{1+\alpha }{1-\alpha }}l_{\alpha }^{\frac{1+\alpha }{1-\alpha }}\;.$$

Therefore, in the α-affine coordinate system $\left\{\theta^{1},\ldots ,\theta^{n}\right\}$, the components of the metric tensor (33) are given by:

$$\begin{array}{ccc} g_{ij}^{\alpha }\left(\theta \right) & = & \mathrm{Tr}\left(\frac{\partial \;l_{\alpha }}{\partial \theta^{i}}\;\frac{\partial \;l_{-\alpha }}{\partial \theta^{j}}\right)\\ & = & \frac{2}{1-\alpha }{\left(\frac{1-\alpha }{2}\right)}^{\frac{1+\alpha }{1-\alpha }}\;\mathrm{Tr}\left(\frac{\partial \;l_{\alpha }}{\partial \theta^{i}}\;l_{\alpha }^{\frac{2\alpha }{1-\alpha }}\frac{\partial \;l_{\alpha }}{\partial \theta^{j}}\right)\;. \end{array}$$

The quantum dually flat structure $\left(g^{\alpha },\nabla^{\alpha },\nabla^{-\alpha }\right)$ for the manifold of positive definite Hermitian operators (or quantum operators) so far described can be also obtained through Equations (3) and (4) when the following divergence is considered,

(36) $$D^{\left(\alpha \right)}\left(\rho_{1},\rho_{2}\right):=\frac{4}{1-\alpha^{2}}\mathrm{Tr}\left(\frac{1-\alpha }{2}\;\rho_{1}+\frac{1+\alpha }{2}\;\rho_{2}-\rho_{1}^{\frac{1-\alpha }{2}}\rho_{2}^{\frac{1+\alpha }{2}}\right)\;,\;\rho_{1},\rho_{2}\in M_{+}$$

This function is called the quantum α-divergence, and it has been introduced on $M_{+}$ as the generalization of the α-divergence (18) of the positive measures [36]. Carrying on this line of reasoning, we can introduce a q-divergence on the manifold of positive Hermitian operators, as well. In analogy with the classical case, we can set α=1−2q in the argument of the trace Tr in Equation (36), and then, we can write the following expression,

(37) $$D_{q}\left(\rho_{1},\rho_{2}\right):=\frac{1}{1-q}\;\mathrm{Tr}\left[q\;\rho_{1}+\left(1-q\right)\;\rho_{2}-\rho_{1}^{q}\;\rho_{2}^{1-q}\right]\;,\;\rho_{1},\rho_{2}\in M_{+}\;,$$

which corresponds to the quantum α-divergence up to the same scalar factor as in the classical case. It is worth noting that this function is different from the extensions of the Tsallis relative entropy to the positive operators in the literature. For example, in [37], the quantum q-divergence was introduced in the following way,

(38) $${\tilde{D}}_{q}\left(\rho_{1},\rho_{2}\right):=\frac{\mathrm{Tr}\left[\rho_{1}\right]-\mathrm{Tr}\left[\rho_{1}^{q}\;\rho_{2}^{1-q}\right]}{1-q}\;,\;\rho_{1},\rho_{2}\in M_{+}\;,$$

for all q∈[0,1). However, both functions, $D_{q}$ and ${\tilde{D}}_{q}$, reduce to the Tsallis relative entropy when restricted to the manifold S of density operators,

$$D_{q}\left(\rho_{1},\rho_{2}\right):=\frac{1-\mathrm{Tr}\left[\rho_{1}^{q}\;\rho_{2}^{1-q}\right]}{1-q}\;,\;\rho_{1},\rho_{2}\in S\;.$$

Analogously, we can restrict the quantum α-divergence (36) to the set of density operators, and since $\mathrm{Tr}\;\rho_{1}=\mathrm{Tr}\;\rho_{2}=1$, we can easily verify that (36) becomes:

(39) $$D^{\left(\alpha \right)}\left(\rho_{1},\rho_{2}\right)=\frac{4}{1-\alpha^{2}}\left(1-\mathrm{Tr}\left[\rho_{1}^{\frac{1-\alpha }{2}}\;\rho_{2}^{\frac{1+\alpha }{2}}\right]\right)\;,\;\rho_{1},\rho_{2}\in S\;.$$

Again, we can see that, on the manifold of density operators, the quantum α-divergence coincides (up to a scalar factor) with the Tsallis relative entropy when we set α=1−2q. The quantum α-divergence (39) was introduced and studied by Hasegawa in [32], and it turns out to be a continuous function of the parameter α. In particular, we have that:

$$\lim_{\alpha \to -1}D^{\left(\alpha \right)}\left(\rho_{1},\rho_{2}\right)=\mathrm{Tr}\left[\rho_{1}\left(\log\rho_{1}-\log\rho_{2}\right)\right]\;,$$

that is the quantum relative entropy $D(\rho_{1},\rho_{2})$ as previously given in Equation (11). Furthermore, the limit α→+1 gives $\lim_{\alpha \to +1}D^{\left(\alpha \right)}\left(\rho_{1},\rho_{2}\right)=D\left(\rho_{2},\rho_{1}\right)$.

At this point, we have in our hands all the ingredients necessary to compute the canonical divergence defined in (6) between $\rho_{1}$ and $\rho_{2}$ on the manifold $M_{+}$ of positive definite Hermitian operators,

(40) $$D\left(\rho_{1},\rho_{2}\right):=\int_{0}^{1}\;t\;{∥{\dot{\gamma }}_{\alpha }\left(t\right)∥}_{\gamma_{\alpha }\left(t\right)}^{2}\;dt\;,$$

where $\gamma_{\alpha }\left(t\right)$ is the $\nabla^{\alpha }$-geodesic from $\rho_{1}$ to $\rho_{2}$. In the $\nabla^{\alpha }$-affine coordinates $\left\{\theta^{1},\ldots ,\theta^{N}\right\}$, this reads as in Equation (32). Here, the norm ${∥\cdot ∥}^{2}$ is induced by the WYD metric given in Equation (33). Hence, in this case, the canonical divergence (40) can be written as:

(41) $$D\left(\rho_{1},\rho_{2}\right)=\int_{0}^{1}t\;\mathrm{Tr}\left({\left({\dot{\gamma }}_{\alpha }\left(t\right)\right)}^{\left(\alpha \right)}\;{\left({\dot{\gamma }}_{\alpha }\left(t\right)\right)}^{\left(-\alpha \right)}\right)\;dt\;,$$

where ${\left({\dot{\gamma }}_{\alpha }\left(t\right)\right)}^{\left(\alpha \right)}$ and ${\left({\dot{\gamma }}_{\alpha }\left(t\right)\right)}^{\left(-\alpha \right)}$ denote the (α) and the (−α) representations of $\dot{\gamma }\left(t\right)$. In order to compute the α representations of $\dot{\gamma }\left(t\right)$, we consider Equation (29) together with Equations (34) and (35). This yields:

$$\begin{array}{ccc} {\left({\dot{\gamma }}_{\alpha }\left(t\right)\right)}^{\left(\alpha \right)} & = & {\dot{\gamma }}_{\alpha }\left(t\right)\\ {\left({\dot{\gamma }}_{\alpha }\left(t\right)\right)}^{\left(-\alpha \right)} & = & \frac{2}{1-\alpha }{\left(\frac{1-\alpha }{2}\right)}^{\frac{1+\alpha }{1-\alpha }}\frac{1+\alpha }{1-\alpha }\;{\left(\gamma_{\alpha }\left(t\right)\right)}^{\frac{2\alpha }{1-\alpha }}{\dot{\gamma }}_{\alpha }\left(t\right)\\ & = & {\left(\frac{1-\alpha }{2}\right)}^{\frac{2\alpha }{1-\alpha }}\;{\left(\gamma_{\alpha }\left(t\right)\right)}^{\frac{2\alpha }{1-\alpha }}{\dot{\gamma }}_{\alpha }\left(t\right)\;. \end{array}$$

We can plug these expressions in Equation (41). Hence, by performing an integration by parts, we get:

$$\begin{array}{ccc} D(\rho_{1},\rho_{2}) & = & {\left(\frac{1-\alpha }{2}\right)}^{\frac{2\alpha }{1-\alpha }}\int_{0}^{1}t\;\mathrm{Tr}\left({\dot{\gamma }}_{\alpha }\left(t\right){\left(\gamma_{\alpha }\left(t\right)\right)}^{\frac{2\alpha }{1-\alpha }}{\dot{\gamma }}_{\alpha }\left(t\right)\right)\;dt\\ & = & {\left(\frac{1-\alpha }{2}\right)}^{\frac{2\alpha }{1-\alpha }}\mathrm{Tr}\left(\int_{0}^{1}t\;{\dot{\gamma }}_{\alpha }\left(t\right){\left(\gamma_{\alpha }\left(t\right)\right)}^{\frac{2\alpha }{1-\alpha }}{\dot{\gamma }}_{\alpha }\left(t\right)\;dt\right)\\ & = & {\left(\frac{1-\alpha }{2}\right)}^{\frac{2\alpha }{1-\alpha }}\mathrm{Tr}\left({\left[t{\dot{\gamma }}_{\alpha }\left(t\right)\frac{1-\alpha }{\alpha +1}{\left(\gamma_{\alpha }\left(t\right)\right)}^{\frac{1+\alpha }{1-\alpha }}\right]}_{0}^{1}-\frac{1-\alpha }{\alpha +1}\int_{0}^{1}{\dot{\gamma }}_{\alpha }\left(t\right){\left(\gamma_{\alpha }\left(t\right)\right)}^{\frac{1+\alpha }{1-\alpha }}\;dt\right)\\ & = & {\left(\frac{1-\alpha }{2}\right)}^{\frac{2\alpha }{1-\alpha }}\mathrm{Tr}\left({\left[t{\dot{\gamma }}_{\alpha }\left(t\right)\frac{1-\alpha }{1+\alpha }{\left(\gamma_{\alpha }\left(t\right)\right)}^{\frac{1+\alpha }{1-\alpha }}\right]}_{0}^{1}-\frac{1-\alpha }{1+\alpha }\frac{1-\alpha }{2}{\left[{\left(\gamma_{\alpha }\left(t\right)\right)}^{\frac{2}{1-\alpha }}\right]}_{0}^{1}\right)\;. \end{array}$$

At this point, we can use Equation (32) to obtain:

$$\begin{array}{ccc} D(\rho_{1},\rho_{2}) & = & {\left(\frac{1-\alpha }{2}\right)}^{\frac{2\alpha }{1-\alpha }}\mathrm{Tr}(\frac{2}{1-\alpha }\left(\rho_{2}^{\frac{1-\alpha }{2}}-\rho_{1}^{\frac{1-\alpha }{2}}\right)\frac{1-\alpha }{1+\alpha }{\left(\frac{2}{1-\alpha }\right)}^{\frac{1+\alpha }{1-\alpha }}\rho_{2}^{\frac{1-\alpha }{2}\frac{1+\alpha }{1-\alpha }}\\ & & -\frac{1-\alpha }{1+\alpha }\frac{1-\alpha }{2}{\left(\frac{2}{1-\alpha }\right)}^{\frac{2}{1-\alpha }}\left(\rho_{2}^{\frac{1-\alpha }{2}\frac{2}{1-\alpha }}-\rho_{1}^{\frac{1-\alpha }{2}\frac{2}{1-\alpha }}\right))\\ & = & \frac{4}{1-\alpha^{2}}\mathrm{Tr}\left(\left(\rho_{2}^{\frac{1-\alpha }{2}}-\rho_{1}^{\frac{1-\alpha }{2}}\right)\rho_{2}^{\frac{1+\alpha }{2}}\right)-\frac{1-\alpha }{1+\alpha }\frac{2}{1-\alpha }\mathrm{Tr}\left(\rho_{2}-\rho_{1}\right)\\ & = & \frac{4}{1-\alpha^{2}}\mathrm{Tr}\rho_{2}-\frac{2}{1+\alpha }\mathrm{Tr}\rho_{2}+\frac{2}{1+\alpha }\mathrm{Tr}\rho_{1}-\frac{4}{1-\alpha^{2}}\mathrm{Tr}\left(\rho_{1}^{\frac{1-\alpha }{2}}\rho_{2}^{\frac{1+\alpha }{2}}\right)\\ & = & \frac{2}{1+\alpha }\mathrm{Tr}\rho_{1}+\frac{2}{1-\alpha }\mathrm{Tr}\rho_{2}-\frac{4}{1-\alpha^{2}}\mathrm{Tr}\left(\rho_{1}^{\frac{1-\alpha }{2}}\rho_{2}^{\frac{1+\alpha }{2}}\right)\;. \end{array}$$

Finally, we can conclude that:

(42) $$D\left(\rho_{1},\rho_{2}\right)=\frac{4}{1-\alpha^{2}}\mathrm{Tr}\left(\frac{1-\alpha }{2}\;\rho_{1}+\frac{1+\alpha }{2}\;\rho_{2}-\rho_{1}^{\frac{1-\alpha }{2}}\rho_{2}^{\frac{1+\alpha }{2}}\right)\;,\;\rho_{1},\;\rho_{2}\;\in M_{+}\;,$$

which corresponds to the α-canonical divergence on the manifold of positive definite matrices.

## 5. Conclusions

The present article is a follow-up of the recently published paper [24]. In the latter one, the authors showed that the canonical divergence defined in Equation (6) provides a powerful tool for unifying classical and quantum information geometry. In particular, such a divergence was proven to coincide with the Kullback-Leibler divergence on the simplex of probability distributions and with the quantum relative entropy on the manifold of quantum states. The effectiveness of the Kullback-Leibler divergence was ascribed in the context of complex systems for quantifying how much a probability measure deviates from the non-interacting states that are modeled by exponential families of probabilities [11]. On the other hand, the quantum relative entropy turned out to be relevant for providing a measure of the many-party correlations of a quantum state from a Gibbs family [13], which in turn was related to the entanglement of quantum systems as defined in [15].

The α-divergence (18) is a generalization of the relative entropy (10). Moreover, the flat α-geometry induced by the α-divergence on the manifold of positive measures constitutes a generalization of the dually flat structure given by the Fisher metric and the mixture and exponential connections. Very remarkably, the α-geometry covers the geometry of q-entropy physics [19]. This bridges a very nice connection between the α-geometry and the generalized statistical mechanics established by Tsallis [17,18]. On the quantum side, the α-divergence (36) was introduced as the generalization of the α-divergence (18) of the positive measures [36]. The quantum α-geometry of the manifold of positive definite matrices is flat, as well, and turns out to be a generalization of the quantum geometry given by the quantum Fisher metric and the quantum mixture and exponential connections induced on the manifold of quantum states by the BKM inner product [16]. In the quantum setting as well, the connection between the α-geometry and the q-entropy physics by Tsallis provides a physical interpretation of the α-divergence. Indeed, a conditional form of the q-entropy was employed for the exact calculation, on some systems, of the separable-entangled separatrix [17].

In this article, we computed the canonical divergence (6) for flat α-connections on the manifold of positive measures, as well as on the manifold of positive definite Hermitian operators. Therefore, we proved that the divergence introduced by Ay and Amari in [21] reduces to the classical α-divergence (18) and to the quantum α-divergence (36). Actually, the equivalence between the canonical divergence (6) and the classical α-divergence was primarily shown in [21]. There, the derivation of $D^{\left(\alpha \right)}$ was grounded on the idea of a squared distance function associated to the α-connections through the vector field $X_{t}\left(p\right)$ given in Equation (5). However, the α-divergence $D^{\left(\alpha \right)}$ does not share all the properties of a squared distance function, unless α=0. For this reason, in the present paper, we obtained the equivalence between D and $D^{\left(\alpha \right)}$ in a different way, by considering the α-divergence as a function of a more general structure.

The present paper is conceived within a project that aims to characterize a general canonical divergence for a given dualistic structure $\left(g,\nabla ,\nabla^{*}\right)$ of a smooth manifold M. This project started with the work by Ay and Amari [21] and later developed in [3], where the concept of the “canonical divergence” was clearly depicted. A considerable effort towards the definition of a general canonical divergence was put forward in [38], where a divergence function was introduced through an extensive investigation of the geodesic geometry of the dualistic structure $\left(g,\nabla ,\nabla^{*}\right)$. Actually, this latter divergence turns out to coincide with the divergence (6) when the dualistic structure $\left(g,\nabla ,\nabla^{*}\right)$ is flat. Further work around this topic was presented in [39], where the very recent divergence was compared with other divergence functions present in the literature. It is commonly accepted that the α-divergence on the simplex is obtained by restricting (18) to the set of normalized positive measures (see [1,6] for more details). Then, the α-divergence of probability distributions in the simplex reads as follows,

(43) $$D^{\left(\alpha \right)}\left(p,q\right)=\sum_{i=1}^{n}\left(1-\frac{4}{1-\alpha^{2}}p_{i}^{\frac{1-\alpha }{2}}\;q_{i}^{\frac{1+\alpha }{2}}\right)\;.$$

One can easily verify that for α=0, this divergence is closely related to the Hellinger distance, which is the distance in the ambient space $M_{+}$ [3]. However, when α=0, the α-connection is the Levi–Civita connection of the Fisher metric. Therefore, in this case, the canonical divergence has to be one half the square of the Fisher distance, as discussed in Section 2, which is different from $D^{\left(0\right)}$ [3]. It would be interesting to evaluate the very recent divergence introduced in [38] on the simplex of probability distributions, which is not α-flat, and to compare it with the α-divergence (43).

On the quantum side, an intriguing work within the above-mentioned project is to consider that very recent divergence function, introduced in [38], on the manifold of pure quantum states, where a dually flat structure does not exist [23]. This will constitute the object of study of a forthcoming investigation.
